# Family factors contribute to mental health conditions – a systematic review

**DOI:** 10.1093/eurpub/ckac129.454

**Published:** 2022-10-25

**Authors:** S Marth, N Cook, P Bain, J Lindert

**Affiliations:** 1 Department of Social Work and Health, University of Applied Sciences Emden/Leer, Emden/Leer, Germany; 2 NHS Foundation Trust, Leeds and York Partnerships, Leeds/York, UK; 3 Countway Library of Medicine, Harvard Medical School, Boston, USA; 4 Brandeis University, Waltham, USA

## Abstract

**Background:**

Family functioning can have positive and negative mental health consequences. Positive relationships can boost mental health, the opposite is true for negative relationships. 1 in 4 individuals are affected by at least one mental health condition in their life. Family-based interventions can help prevent the onset of mental health conditions and mitigate its consequences.

**Methods:**

Following databases were systematically searched: Medline; PsychInfo, Web of Sciences and Cochrane, resulting in 3719 hits. After removing 12 duplicates, 3707 studies were screened. After exclusion of irrelevant studies, 362 studies were assessed for eligibility and 40 studies were included. Inclusion criteria were original studies with ≥100 participants, ≥18 years, general population, and family members. Exposure had to be family social cohesion or conflict, or social capital. The outcome had to be a mental health condition.

**Results:**

Most studies (n = 37) used a cross-sectional design. 37 studies included a measure of family functioning and 3 studies used one of family structure. Most used was the Family Adaptability and Cohesion Evaluation Scale (n = 17), followed by the Family Functioning Scale (n = 5). Family relationship quality was related to depression, anxiety, and substance use. All aspects family cohesion were related to mental health outcomes. Family conflicts are associated with an increase in mental health conditions.

**Conclusions:**

Family cohesion shows an association with positive mental health while conflict is associated with negative mental health. This is an indication, that interventions at the family level are useful to help prevent/mitigate mental health conditions over the life course. Main message: As mental health conditions are a big public health issue affecting at least 1 in 4 individuals, family-based interventions for mental health condition prevention could not only help individuals but the whole family to strengthen and maintain positive mental health.

